# Field Efficacy of Anticoagulant Rodenticide Towards Managing Rodent Pests in Jitra Rice Field, Kedah, Malaysia

**DOI:** 10.21315/tlsr2024.35.3.11

**Published:** 2024-10-07

**Authors:** Maisarah Burhanuddin, Hafidzi Mohd Noor, Hasber Salim, Nur Athirah Asrif, Syari Jamian, Badrul Azhar

**Affiliations:** 1 Department of Plant Protection, Faculty of Agriculture, Universiti Putra Malaysia, 43400 Serdang, Selangor, Malaysia; 2 School of Biological Sciences, Universiti Sains Malaysia, 11800 USM Pulau Pinang, Malaysia; 3 Research and Development Department, Eco-Management Unit, Wilmar Plantations S/B, Locked Bag 34, 90009 Sandakan, Sabah, Malaysia; 4 Laboratory of Climate-Smart Food Crop Production, Institute of Tropical Agriculture and Food Security (ITAFoS), Universiti Putra Malaysia, 43400 Serdang, Selangor, Malaysia; 5 School of Environmental and Geographical Sciences, University of Nottingham Malaysia, 43500 Semenyih, Selangor, Malaysia; 6 Biodiversity Unit, Institute of Bioscience, Universiti Putra Malaysia, 43400 Serdang, Selangor, Malaysia

**Keywords:** Chlorophacinone, Flucoumafen, Rice Field, Rodent Pest, Bandicota indica, Rattus argentiventer, *Rattus rattus*, *Suncus murinus*, Klorofasinon, Flukoumafen, Sawah Padi, Tikus Perosak, *Bandicota indica*, *Rattus argentiventer*, *Rattus rattus*, *Suncus murinus*

## Abstract

Frequent encounters with the greater bandicoot rats (*Bandicota indica*) following high rodent damage towards rice crops and lack of information on the species had encouraged this study to be conducted to test the relevance of using first- and second-generation rodenticide in a field efficacy test. This study also attempts to detect any sign of resistance of current rodent pest populations towards chlorophacinone (0.005%) and flucoumafen (0.05%) for the control of field rats predominant rice field agrosystem of the Kedah in northern peninsular Malaysia. Six different treatments over dry and wet rice planting season together with trapping exercise. The observation was evaluated based on the number of active burrows, counting tiller damage due to rodent attack and trapping index. The results indicated that flucoumafen gives better rodent control and has a better impact (*p* < 0.05) although chlorophacinone is still relevant to be applied (*p* < 0.05). Treatments during the off-planting season (September–February) are more effective compared to the main planting season (March–August). Rodent control during the early off-planting season is encouraged for better rodent management in the rice field and the use of bait stations to increase the weatherability of the baits.

## Abstract (fr)

**Abstrak:**

Laporan petani mengenai pertembungan yang kerap dengan tikus ekor pendek (*Bandicota indica)*, dan kerosakan tikus yang tinggi terhadap tanaman padi, serta kekurangan maklumat mengenai spesies ini telah mendorong kajian ini dijalankan untuk menguji relevansi penggunaan racun rodentia generasi pertama dan kedua dalam ujian keberkesanan lapangan. Kajian ini berusaha untuk mengesan sebarang tanda ketahanan populasi perosak tikus semasa terhadap klorofasinon (0.005%) dan flukoumafen (0.05%) untuk mengawal tikus di agroekosistem padi yang dominan di Kedah, Malaysia. Enam rawatan berbeza telah dijalankan sepanjang musim penanaman padi kering (musim luar penanaman) dan basah (musim utama penanaman) bersama dengan perangkap fizikal. Pemerhatian dinilai berdasarkan bilangan lubang aktif, mengira kerosakan batang padi akibat serangan tikus, dan indeks perangkap. Hasil menunjukkan bahawa flukoumafen memberikan kawalan tikus yang lebih baik dan mempunyai impak yang lebih baik (*p* < 0.05) walaupun klorofasinon masih relevan untuk digunakan (*p* < 0.05). Rawatan semasa musim luar penanaman (September–Februari) adalah lebih berkesan berbanding dengan musim penanaman utama (Mac–Ogos). Kawalan tikus semasa awal musim luar penanaman digalakkan untuk pengurusan tikus yang lebih baik di ladang padi dan penggunaan stesen umpan untuk meningkatkan ketahanan umpan terhadap cuaca.

**Highlights**
- Four different small mammal species were found co-habituate Jitra rice field which were the greater bandicoot rat (*Bandicota indica*), the ricefield rat (*Rattus argentiventer*), the black rat (*Rattus rattus*) and one shrew species, the Asian house shrew (*Suncus murinus*).
- Usage chlorophacinone is still relevant however flucoumafen application need to applied sensibly and the treatments application during the off-planting season (April–September) was found to be more effective compared to the main planting season (October–February).
- Rodent control during the early off-planting season is encouraged for better rodent management in the rice field and the use of bait stations to increase the weatherability of the baits.

## INTRODUCTION

Bandicoot rats are one of the largest commensal rodent pest species and can invade both the urban and agro-ecosystem (Aplin *et al*. 2016; Shukor *et al*. 2018*)*. They were found to feed on various crops and storage products (Brooks & Htun 1978; Brooks *et al*. 1980; Dhar & Singla 2014; Hossain & Khalequzzaman 2002; Hussain *et al*. 2016; Mehmood *et al*. 2011). Bandicoot rats were nocturnal, subterranean and found to feed on various food such as plant materials, mollusks, crustaceans and earthworms (Lim 2015). *Bandicota* sp. may feed primarily on invertebrates when they are at low population densities. However, they were reported to cause heavy damage at high densities in various agriculture fields. In rice fields in India, the damage is evident when patches in the centre of the field started to appear. It starts shortly after sowing and continues through panicle initiation, milky stage, panicle development stage and ripening stage (Borah & Mallick 2016). According to Sarwar (2015), bandicoot rat damages stems, flowers and grains of rice drop and cause damage in three phases in the rice growing stages.

They can be active at all hours. During the day, they spent time extending their burrows (Hossain & Khalequzzaman 2002; Sagar & Bindra 1978). These burrows in banks and under rail tracks may result in indirect damage: derailment of trains, loss of irrigation water or flooding. At night, the activity is confined to collecting and storing food. A single burrow has been found to contain 7.3 kg of wheat heads (Sagar & Bindra 1978), and there are many variations in characteristics of the burrow system built by the bandicoot rats; presumably related to the nature of crops, seasons, soils and moisture content (Hussain *et al*. 2016).

As for control measures, several rodenticides have been tested and reported. First and second-generation rodenticides have been used in India, Burma and Bangladesh (Brooks & Htun 1978; Parshad & Chopra 1986; Sheiker & Jain 2008; Sridhar *et al*. 2015; Borah & Mallick 2016). Other non-anticoagulant rodenticides such as bromethalin (Brooks *et al*. 1980; Khan & Rizvi 2000) and zinc phosphide (Htun & Brooks 1979; Singla & Parshad 2010). Recently, researchers look into the potential usage of plant extracts (Singla & Garg 2013; Kaur *et al*. 2022) and the synergistic reaction between rodenticides (Naseri & Zohdi 2011).

Physical interventions and the use of rodenticides can be particularly difficult to implement, due to difficulties intrinsic to rodent physiology and behavioural adaptations. Most pest rodent species show signs of neophobia (Ennaceur *et al*. 2008; Modlinska *et al*. 2015; Raab *et al*. 2018; Witmer *et al*. 2020; Vicente & De la Casa 2021), both towards novel objects and tastes, which results in high levels of “trap-shyness” (Weihong *et al*. 1999; Herawati & Purnawan 2019), and low bait acceptance (Brunton *et al*. 1993; Shumake *et al*. 2002; Bedoya-Perez *et al*. 2021). Rodents also learn from the experience of conspecifics; if conspecifics emit signals of distress, e.g., getting caught in a trap as they are less likely to approach the same area later on (Brudzynski & Chiu 1995; Brudzynski 2009; Haapakoski *et al*. 2018). Furthermore, the widespread use of rodenticides has induced the development of resistance in rodent populations to first and second-generation anticoagulants (Lund 1984; Quy *et al*. 1998; Buckle *et al*. 1994; Marquez *et al*. 2019; Blažić *et al*. 2020; McGee *et al*. 2020).

Efficacy tests are one of the field trials that commonly be conducted to evaluate the suitability of the rodenticide or any rodent control strategies before applied in the field (Tongtavee 1980; Kaudeinen & Rampaud 1986; Hoque & Olvida 1988; Parshad 2002; Pitt *et al*. 2010; Horak *et al*. 2020; Rizo *et al*. 2006; Motro *et al*. 2019; Frankova *et al*. 2019; Krijger *et al*. 2020). Substances such as zinc phosphide, bromadiolone, cholecalciferol and many more were usually applied to manage rodent pests from causing severe damage to crops. Other studies on efficacy field trials were mentioned in Table 1.

According to local farmers in Jitra, despite using shelf available rodenticides and applying it in accordance with authority recommendations, their encounter with the greater bandicoot rat, *Bandicota indica* was still frequent and, in some areas, the rodent damage is high. Therefore, this field trial was conducted to evaluate the relevance of using anticoagulant rodenticides to control rodent pest attack in the rice field, especially in areas reported with high incidence of the greater bandicoot rats. We are also exploring whether the usage of anticoagulant rodenticides in this area still appropriate or does the population finally shows resistant after 80 years of introduction. The objective of this study was to investigate field efficacy of anticoagulant rodenticides for the control of field rodents infesting rice crops. It was hypothesised that these rodenticide baits are still relevant to be used for rodent pest management.

## MATERIALS AND METHODS

### Study Site

The study was conducted in farmer’s field of Kampung Raja and Kampung Ketol in Jitra, Kedah (6°18′56.6″N 100°21′07.6″E) from May 2019 till February 2020. Six study sites were selected to survey the rodent species infestations reported by local farmers. The habitat of the study area is a tropical scrub vegetation and cultivation of rice field crops. The western and southern part of the area is dominated by a rice field area and human settlements. There is a reserve forests namely Bukit Wang Forest Reserve located northeast from the study site. There are two planting seasons per year; dry months (April–September) and monsoonal season (October–February). In the drier month, usually there is limited water source for agriculture purposes, thus rice planting activities are put at halt during this period and or be carried out using direct seeding method, without relying on water logging as rice weed control.

All these three areas have nearly the identical rice planting season; two seasons per year. Ploughing usually starts during March/April and August/September. However, instead of the normal wet and dry planting season like in Perak, there are prominent few months of dry season during early dry planting season in Kedah and Perlis. Rice planting during these times will highly depend on water supply from Muda Dam. If the dam capacity is less than 45%, farmers will be recommended by authorities resort to dry sowing method.

### Study Design

The size of each selected experimental plot was 0.2 ha with three replications for each six treatments and one control sites (i.e., No rodenticide application) with contiguous field crops. The sites were chosen after consultation with local farming individuals and having historically low rodent infestation until 2017, where they reported frequent encounter with *B. indica* and higher rodent damage was reported especially during booting and harvesting stage of the rice crop. The treated and reference/control sites were selected at least 200 m apart keeping in view to prevent dispersal of rodents between sites (Munawar *et al*. 2020). The baits were left and checked after 72 h and any remaining baits were measured, and the amount of bait were reset (Singla *et al*. 2023). In the selected sampling sites numerous active rodent burrows were observed inside the croplands and at field boundaries, with fresh digging and distinctive damage patterns to the surrounding crop plants. The greater bandicoot rat (*B. indica*) and the rice field rat (*R. argentiventer*) were assumed to be the main rodent pests at the time of treatments.

### Bait Preparation

For comparative evaluation, two rodenticides namely 0.05% chlorophacinone (a first-generation anticoagulant), and 0.005% flucoumafen (a second-generation anticoagulant) were used in this study. Flucoumafen was readymade in a ready-to-use form and already contain attractant within, however, chrophacinone comes in a liquid form. Recommendation method by the manufacturer was to mix the bait for 30 min minimum and apply them straight onto the bunds as it has excellent weather durability. Nevertheless, in this study, 250 mL chlorophacinone was mixed with 10 kg unhusked rice for overnight before applied in the field using bait station.

The baiting was continued for 1 week with inspection every 3 days. Chlorophacinone and flocoumafen were used as baits using similar dosage as trials in the laboratory; 30 g of chlorophacinone (0.05%) mixed with unhulled rice, and 18 g of flucoumafen (0.005%) or 5 tablets were used per bait station. The weight of the bait was recorded and replaced for the amount of bait consumed.

Treatments A and B, rice plots were treated using during land preparation stage and during harvesting stage (Table 2). Treatments C and D adopted conventional control, practiced by local farmer practice which apply the rodenticides during harvesting stage or after signs of rodent attack was observed in their plots (Table 2). Plots in Treatment E only relying on single door spring trap, which was also a farmers practice if they cannot afford to purchase any rodenticides. No action was taken in Treatment Control F and it serve as control plot (Table 2).

### Bait Placement Technique

**Figure 1:**
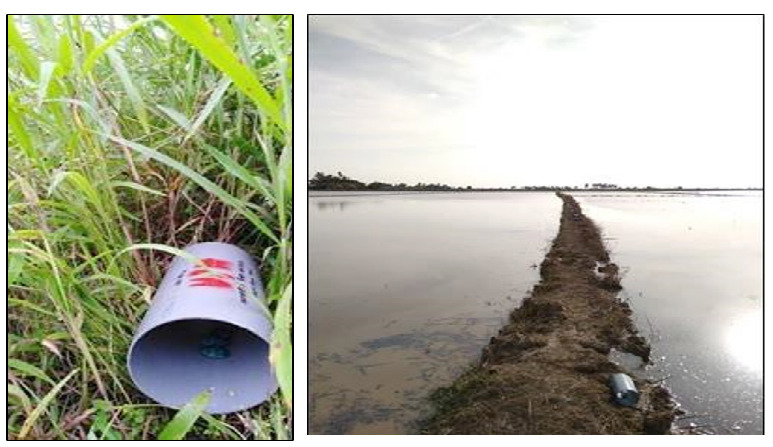
Bait stations placed nearby burrow entrances close to shrubs (left). Bait station placed on bunds during land preparation stage (right).

Baiting was done in the rice field crop at two growth stages (germination and maturity). The procedure used was the same for both crops. The bait was presented in a polyvinyl chloride (PVC) tube of 6.5 cm radius and 30 cm length tube (Fig. 1). During each visit the remaining bait was weighed using automatic weight measurement. Damp or soiled bait was replaced at each visit (Hussain & Prescott 2006; Munawar *et al*. 2020). Each bait station (30 unit/treatment) was regarded as a replicate for the experiment/treatment (Munawar *et al*. 2020).

### Tiller Damage Incidence

Evaluation on active burrow count, tiller damage and trapped rodent were measured in all sites during harvesting stage to see any differences between treatments. Percentage of cut tillers were recorded using 30 cm × 30 cm frame. Replicated randomly 25 times/plot (Borah & Mallick 2016; Borah 2021).


$$\text{Tiller damage incidence }(p)=\frac{A\times 100}{A+B}$$

where *A* = total number of tillers in the 25-hill sample and *B* = total number of healthy tillers in 25-hill sample.

### Live Burrow Count (LBC/ha)

Rodent burrows can be found along the waterways, bunds and by the roadside nearby rice fields. Active burrows surrounding the treatment plots were counted by closing the entrances with soil and vegetation and were inspected on the following day. Any clearance on the entrances indicated an active burrow. Live burrow count was calculated during land preparations stage (before treatment) and during harvesting. The estimated mortality index or percentages of reduction in the rat’s population were calculated using the following formula (Borah & Mallick 2016; Munawar *et al*. 2020; Borah 2021).


$$\%\;\text{success}=\frac{a-b}{a}\times 100$$

where *a* = pre-treatment burrow census and *b* = post-treatment burrow census.

### Trapping Index

Kill trapping (Sheiker & Jain 2008; Htwe *et al*. 2021) was conducted for two cropping seasons (a dry season and a wet season) from May 2019 till February 2020. To collect animal samples of both *B. indica* and *R*. argentiventer, steel live traps were set at each rice growing stage. They were set late in the evening (1800) and checked in the early morning (0730). Traps were usually set for 2 to 4 consecutive nights in one place and then moved to another place (within 50 m) in the same location if few rats were caught (Htwe *et al*. 2021; Sheiker & Jain 2008; Islam & Karim 1995). The traps were also covered to avoid any stealing and trapping unwanted animals (Weihong *et al*. 1999).

Traps were set along the irrigation channel, creek line and levees around the rice field. Trapped rats were weighed (g) and measured for body length from tip of the nose to middle of the anus (mm), tail length from middle of the anus to the tip of the tail (mm) and ear length from bottom of ear notch to the furthest point along the rim (mm). Morphological measurements were used to confirm the identity of the species (Htwe *et al*. 2021; Borah & Mallick 2016; Borah 2021). Rodent Trapping Index and control success (%) for trapping index (Chaudhary & Tripathi 2009) were calculated using these formulas:


$$\text{Index of rodent abundance}=\frac{\text{Number of rat caught in all traps}}{\text{Number of traps}\times \text{night}}$$

### Statistical Analysis

Descriptive statistics were performed to compute means and standard errors (±) of the data. After data arrangement, the significant difference was determined whether post-treatment efficacy values differed from each other in different crops stages and study sites (treated/reference) by using ANOVA. The least significant difference (LSD) test was applied to assess the significant differences among means. LSD test is demonstrating the significant differences between the mean values of different treatments at 5% level of significance (Munawar *et al*. 2020; Borah & Mallick 2016; Borah 2021). All statistical calculations were performed using IBM SPSS Statistics version 25.

## RESULTS

### Species Composition

**Figure 2:**
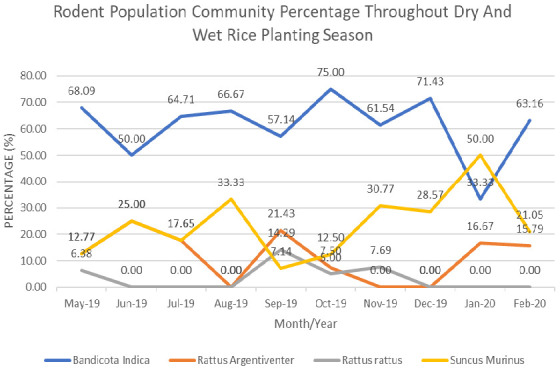
Rodent population percentage throughout dry and wet rice planting season (2019/2020). Percentage of rodent pest composition throughout rice growing stages during dry and wet planting season. Dried fish/shrimp paste was used during dry season, and fresh crab/prawn was used in wet season.

A total of 165 rodents were caught throughout the wet and dry season of year 2019 till early 2020. The number of *B. indica* caught exceeded other rodent species in the study area for all growing stages (Fig. 2) in both dry season [land preparation (68%), vegetative state (50%), reproductive stage (64.71%), ripening stage (66.67%), harvesting stage (57.14%)] and in wet season [land preparation (75%), vegetative state (61.54%), reproductive stage (71.43%), ripening stage (33.33%), harvesting stage (63.16%)]. Other rodent pest species recorded were the rice field rat (*R. argentiventer*), the black rat (*R. rattus*) and the Asian House Shrew (*Suncus murinus*).

### Baiting Treatments

The consumption of 0.005% flucoumafen and 0.05% chlorophacinone ranged from 30%–80% and 30%–60%, respectively (see Table 3). One way ANOVA test calculated that there was a significant difference between chlorophacinone and flucoumafen uptake in Treatment B (*p* < 0.05) in both rice planting seasons compared to other treatments. The 0.005% flucoumafen and 0.05% chlorophacinone were consumed 60%–80% and 45%–60%, respectively during dry season, which was more than wet season.

### Tiller Damage Incidence

Flucoumafen was found to give a better percentage of control success of percentage in tiller damage in both dry season (14.63%) and in wet season (14.74%) when applied in both land preparation and harvesting stage (Table 3). It was also found to be effective up to 12% control if only be applied during harvesting stage (Treatment D) in dry season and in 10% control during wet season compared to Treatment E and control plots.

Chlorophacinone, despite giving a good control in both planting season, it was found to gives a relatively less percentage of control success compared to the flucoumafen (Table 2). Application of chlorophacinone once (CS_trtc, dry_ = 12.48%; CS_trtc, wet_ = 11.13%) during harvesting or twice (CS_trta, dry_ = 13.71%; CS_trta, wet_ = 13.56%) in the field during both season gives a fairly similar result. Combination of chemical control with trapping effort gives a better control rather than relying solely on traps (*p* < 0.05; CS_trte, dry_ = 1.12%; CS_trte, wet_ = 5.12%) (Table 3). Conducting chemical and trapping control in dry season is more effective in reducing tiller damage than during wet season (*p* < 0.05).

### Live Burrow Count (LBC/ha)

Flucoumafen was found to give a better percentage of control success of percentage in live burrow count in both dry season (18.70%) and in wet season (17.74%) when applied in both land preparation and harvesting stage (Table 4). It was also found gives better control in reducing live burrow count compared to solely relying on trapping in both dry and wet season (Fig. 3).

Chlorophacinone, despite giving a good control in both planting season, it was found to gives a relatively less percentage of control success in reducing active burrow count compared to the flucoumafen (Table 4). Application of chlorophacinone once (CS_trtc, dry_ = 16%; CS_trtc, wet_ = 14.25%) during harvesting or twice (CS_trta, dry_ = 4.17%; CS_trta, wet_ = 15.61%) in the field during both season gives a fairly similar result. Combination of chemical control with trapping effort gives a better control rather than relying solely on traps (*p* < 0.05; CS_trte, dry_ = 0.85%; CS_trte, wet_ = 2.50%) (Table 4). Conducting chemical and trapping control in dry season is more effective in reducing number of active burrows than during wet season (*p* < 0.05).

### Trapping Index

For trapping index assessment, flucoumafen was also found to gives a better percentage of control success of percentage in tiller damage in both dry season (87.53%) and in wet season (62.51%) when applied in both land preparation and harvesting stage (Table 5). It was also found to be effective up to 88% control if only be applied during harvesting stage (Treatment D) in dry season and in 57% control during wet season compared to Treatment E and control plots. Application of chlorophacinone once (CS_trtc, dry_ = 71.42%; CS_trtc, wet_ = 16.63%) during harvesting or twice (CS_trta, dry_ = 87.53%; CS_trta, wet_ = 19.89%) in the field during both season gives a fairly similar result.

During dry season, there was no difference in Treatments A, B and D. Applying rodenticides using these treatments may give a similar result in reducing trapping index (*p* < 0.05) (Table 5). However, during wet season, Treatment B gives a better trapping index control success percentage compared to other treatments (*p* < 0.05). Combination of chemical control with trapping effort gives a better control rather than relying solely on traps (*p* < 0.05; CS_trte, dry_ = 66%; CS_trte, wet_ = 20%) (Table 5). Conducting chemical and trapping control in both dry and wet season was effective in reducing trapping index (*p* < 0.05).

## DISCUSSION

Coexistence of rodent pests are common in agricultural lands (Baht & Sujatha 1993; Chaudhary & Tripathi 2009; Motro *et al*. 2019; Singla & Babbar 2015; Htwe *et al*. 2012; Borah 2021) and urban areas (Cavia *et al*. 2009; Oyedele *et al*. 2015; Dammhahn *et al*. 2020; Bedoya-Perez *et al*. 2021). One of the difficulties in rodent management is dealing with multiple rodent species coexisting in one place since small rodent populations have unpredictable dynamics (Andreassen *et al*. 2021). Unexpected epidemics could result from this, affecting the health, conservation and economic sectors. With multiple rodent species in an area, their competition for resources can be perilous especially towards small scale rice farmers. In the Philippines, *R. argentiventer* was found together with *R. tanezumi* and even have similar breeding ecology (Htwe *et al*. 2012) while in Pakistan, Khanam *et al*. (2017) discovers that eight different rodent species; the house mouse (*Mus musculus*), black rat (*R. rattus*), Indian gerbil (*Tatera indica*), soft-furred field rat (*Millardia meltada*), Indian bush rat (*Golunda ellioti*), lesser bandicoot rat (*Bandicota bengalensis*), short-tailed bandicoot rat (*Nesokia indica*) and little Indian field mouse (*Mus booduga*) cohabitate in four rural human villages. The occurrence of multiple rodent species is common, the situation may cause high crop loss, especially in vast agricultural areas.

In this study, flucoumafen baits has the highest bait consumption and resulting in better control of pest by having significantly reduce number of tiller damage, burrow count and trapping index (Tables 3–5). In Indonesia, usage of flucoumafen was proven to reduce seedling damage from 28% down to 0.4% and increase unhauled rice yield as much as 4.8 mt, while in the Philippines, flucoumafen is an excellent rodenticide to control rodent damage towards rice crops (Pearman 1990). Since rodent population here in Jitra, Kedah are still responding well with both rodenticide, usage of first-generation rodenticide should be utilised and incorporated together with ecologically based rodent management (Brown *et al*. 1999; Brown & Khamphoukeo 2009; Ramsay & Wilson 2000; Herawati & Purnawan 2019; Jacob *et al*. 2010; Singleton *et al*. 2004; 2021).

However, second-generation rodenticides should be applied heedfully as they can be very toxic to non-target animals and causing secondary poisoning throughout the food chain (Alomar *et al*. 2018; Rizor *et al*. 2006; Lemus *et al*. 2011). Any development of non-susceptible pest generation should be avoided as they required more potent rodenticide and more comprehensive pest control later in the future. Despite the risk of poisoning, anticoagulant rodenticides are widely used and have a long history of effectiveness. It is likely that it will lead the practice of rodent control in future (Hadler & Buckle 1992), although, current studies start to venture into greener option of rodenticides includes the usage of plant extracts (Boublata *et al*. 2021; Onasanwo *et al*. 2008; Kim *et al*. 2019), fertility control (Imakando *et al*. 2021) and synergistic of toxic compound (Singh *et al*. 2017*;* Eason *et al*. 2020), rather than relying on anticoagulants as the cases of secondary poisoning towards non-target animals are rising (Sanchez-Barbudo *et al*. 2012; Alomar *et al*. 2018; Rizor *et al*. 2006; Lemus *et al*. 2011).

The usage of bait stations seems to improve the rodenticide effect when compared practices by farmers without bait stations. The number of rodents trapped also differs when compared with control plots. This may be due to the more pleasant and attractive smell of the baits. Development of future rodenticide with enjoyable smell to the rodents will improve bait uptake in the rice field, and farmers are encouraged to apply rodenticides within bait stations not only to enhance the rodenticide effects but also to prevent toxicity towards non-target animals.

Each year, farmers must decide whether or not to allocate funds for rodent management. While predicting rodent outbreaks requires complex calculation of various variables (Davis *et al*. 2004), and eruptive rodent population dynamics can be triggered by interannual variation in environmental factors (Stenseth *et al*. 2003). Furthermore, with inflation occurs in Malaysia (Cheng & Tan 2002; Yusof *et al*. 2021), local farmers decision to invest in rodent control can be a huge gamble on the crop yield and profit. Some small-scale farmers may need to resort to cultural control and one of the important elements is good timing.

In this study, we found that during early dry planting season (February–May) is a good time to conduct control towards rodent pest in the rice field. Using chemical control and trapping method resulted in an effective method to manage tiller damage, active burrows and trapping index. Farmers in Kedah are encouraged to utilise the 3-month dry season as a period to controlling rodent pests by setting up traps, clearing lands and apply rodenticide vigilantly. Thitipramote *et al*. (2009) and his team discovered *Bandicoot* sp. reproduced during the rainy season in Southern Thailand, with some reproductive activity extending into the following dry period for a variable length of time. The prolonged dry seasons is a natural barrier that suppress new generation of rodent population emerging with existing population. The high temperature and lack of food supply will naturally suppress the number of rodent survivors for the next planting season. Therefore, with additional control measures taken during this period, rodent attack will surely be under control.

Further research should be done to investigate the usage of safer, natural but potent rodenticide (Boublata *et al*. 2021; Onasanwo *et al*. 2008; Kim *et al*. 2019; Imakando *et al*. 2021), improving baiting technique and boosting numbers of natural predator in the area such applying barn owl propagation programmed or studying other potential predator candidates in effort to build alternatives on natural predator programmes. A breeding study of greater bandicoot rat and rice field rat will also assist in understanding how these species cohabitate and improve control methods towards them.

## CONCLUSION

We discovered that both first- and second-generation rodenticides gives control effects towards rodent population in the studied area in term of reducing tiller damage, active burrows and trapping index. However, usage of fist generation rodenticide is still relevant in controlling rodent pests in these rice fields and application of second-generation rodenticides need to be carefully applied. The area is still in pristine condition as we discover several natural predators around such as Javan mongoose, avian predators, Asian water lizards and even smooth coated otter in the area. Application of the rodenticides need to pe properly distributed using bait station to avoid toxicity towards non-target animals such as these local predators. Furthermore, we noticed that it is harder to capture rats after the tillering rice planting stage. Presumably, due to existence of other food source, the pests do not bother to go for the traps. Therefore, massive rodent pest control (trapping, hunting or baiting) during the prominent dry season before and during land preparation stage is a good timing in managing rodent pest in rice field Jitra. The lack of food source in the field will encourage the rats to forage food from installed traps. These will not only reduce the number of rodent survivals later, but also increase chances to capture rodent pests in the area and can act as an alternative method rather than relying on heavy chemical control during rice growing stage.

## Figures and Tables

**Table 1:** List of various rodenticides andorganiccompoundtestedinfieldtrials from other researchers.

Active ingredients	Rodent species	Trials	Area	Country	Source
Zinc phosphide Bromadiolone	Lesser bandicoot rats, *B. bengalensis* Little Indian field mouse, *Mus booduga* Soft furred rat, *Millardia meltada* Indian bush rat, *Golunda ellioti*	Efficacy test (field)	Sugarcane field	India	Singla & Parshad (2010)
Zinc phosphide Brodifacoum	Lesser bandicoot rats, *B. bengalensis* Roof rat, *Rattus rattus*	Efficacy test (field)	Wheat-groundnut cropping system	Pakistan	Munawar *et al*. (2020)
Bromadiolone, Difenacoum Brodifacoum	Norway rats, *R. norvengicus*	Efficacy test (field)	Farmsteads	England	Buckle *et al*. (2020)
Diphacinone Cholecalciferol (synergistic)	Roof rats, *Rattus rattus*	Efficacy test (laboratory and field)	Shipyards	New Zealand	Eason *et al*. (2020)
Acetylsalicylic Acid (ASA)	Roof rat, *Rattus rattus*	Efficacy test (laboratory and field)	Clothes storage	Egypt	Kandil *et al*. (2022)
Warfarin Bromadiolone Difethialone Brodifacoum	House mouse, *Mus musculus*	Efficacy test (laboratory and field)	Agriculture buildings	Czech Republic	Frankova *et al*. (2019)
Norbormide	Norway rat, *Rattus norvegicus*	Efficacy test (field)	Chicken farms	New Zealand	Shapiro *et al*. (2020)
Zinc phosphide Bromadiolone	Lesser bandicoot rats, *B. bengalensis* Roof rat, *Rattus rattus*	Efficacy test (laboratory and field)	Grain storage godowns	India	Namala (2020)
Zinc phosphide Bromadiolone	Lesser bandicoot rats, *B. bengalensis* Little Indian field mouse, *Mus booduga* Soft furred rat, *Millardia meltada* Indian gerbil, *Tatera indica*	Efficacy test (field)	Wheat, rice and sugarcane field	India	Singla *et al*. (2023)
Brodifacoum-25D	Mice, *Mus* spp. Rats, *rattus* spp.	Efficacy test (laboratory and field)	Island ecosystem	Hawaii	Kappes *et al*. (2022)
EP-1 (Quinestrol(E) +evonorgestrel (P)) Bromadiolone	Multimammate mouse, *Mastomys natalensis*	Efficacy test (field)	Maize field	Zambia	Imakando *et al*. (2021)
Coumatetralyl Chlorophacinone Brodifacoum Flucoumafen Brodifacoum	Malayan wood rat, *Rattus tiomanicus* Rice field rat, *Rattus argentiventer* Malaysian House Rat, *Rattus rattus diardii*	Efficacy test (field)	Oil palm	Malaysia	Mohd Noh *et al. (*2022)
This study: Chlorophacinone Flucoumafen	Greater bandicoot rat, *Bandicota indica* Rice field rat, *Rattus argentiventer*	Efficacy test (field)	Rice field	Malaysia	-

**Table 2:** Treatments for rodent control in rice field.

Label	Treatment	Application
A	Chlorophacinone + trapping	Conducted during land preparation and harvesting stage.
B	Flucoumafen + trapping	Conducted during land preparation and harvesting stage.
C	Chlorophacinone + trapping	Conducted in harvesting stage. Farmer’s practice.
D	Flucoumafen+ trapping	Conducted in harvesting stage. Farmer’s practice.
E	Trapping only	Conducted in harvesting stage.
F	Control	No action taken.

**Table 3:** Percentage of tiller damage before and after treatments fordry and wet rice planting season.

Treatments	Dry planting season				Wet planting season			

	Bait uptake (%)	Percent tiller damage			Bait uptake (%)	Percent tiller damage		
	
		BTC	ATC	% control success		BTC	ATC	% control success
A	60^b^	3.11	2.36	13.71^a^	49^b^	4.28	3.21	13.56
B	80^a^	3.25	2.42	14.63^b^	60^a^	4.52	3.36	14.72
C	45^b^	3.74	2.91	12.48^a^	33^b^	5.24	4.19	11.13
D	60^b^	3.19	2.46	12.92^a^	32^b^	3.99	3.18	10.04
E	-	2.71	2.17	1.12^c^	-	3.28	3.05	7.08
F (control)	-	2.53^a^	2.45	1.13^c^	-	4.00	3.61	5.12
(*α* = 005)				0.0018				0.7191
Sed (±)				7.4091				7.9623
CV				4.18				4.03

*Notes*: BTC = before treatment count; ATC = after treatment count; SED = standard error deviation;

abc = Result of LSD analysis comparing between treatments.

**Table 4:** Live burrow count before and after treatments for dry and wet rice planting season.

Treatments	Dry planting season				Wet planting season			

	Bait uptake (%)	LBC/ha			Bait uptake (%)	LBC/ha		
	
		BTC	ATC	% control success		BTC	ATC	% control success
A	60^b^	4.95	3.55	16.00^a^	49^b^	4.21	3.16	14.25^a^
B	80^a^	5.11	3.50	18.70^a^	60^a^	5.11	3.57	17.74^a^
C	45^b^	5.37	4.94	4.17^b^	33^b^	4.74	3.46	15.61^a^
D	60^b^	5.59	4.93	6.27^b^	32^b^	4.72	3.62	13.19^a^
E	-	5.22	5.31	0.85c	-	4.34	4.23	2.50^a^
F (control)	-	5.39^a^	5.45^a^	1.11	-	4.33	4.31	0.23
*p*-value (*α* = 005)				0.0001				0.3768
Sed (±)				0.2210				0.0589
CV				17.32				2.14

*Notes*: LBC = live burrow count; BTC = before treatment count; ATC = after treatment count; SED = standard error deviation;

abc = result of LSD analysis comparing between treatments.

**Table 5:** Trapping index before and after treatments for dry and wet rice planting season.

Treatments	Dry planting season				Wet planting season			

	Bait uptake (%)	Trapping index			Bait uptake (%)	Trapping index		
	
		BTC	ATC	% control success		BTC	ATC	% control success
A	60^b^	12.67	1.58	87.53^a^	49^b^	7.92	6.34	19.95a
B	80^a^	12.67	1.58	87.53^a^	60^a^	12.67	4.75	62.51^b^
C	45^b^	11.09	3.17	71.42^b^	33^b^	9.50	4.75	50.00^a^
D	60^b^	14.26	1.58	88.92^a^	32^b^	11.09	6.34	42.83^a^
E	-	9.50	3.17	66.63^b^	-	7.92	6.34	19.95^a^
F (control)	-	14.26	12.67	11.15^c^	-	12.67	11.09	12.47^a^
*p*-value (*α* = 005)				0.00				0.02
Sed (±)				3.23				1.79

*Notes:* LBC = live burrow count; BTC = before treatment count; ATC = after treatment count; SED = standard error deviation;

abc = result of LSD analysis comparing between treatments.
